# Time Savings with Rituximab Subcutaneous Injection versus Rituximab Intravenous Infusion: A Time and Motion Study in Eight Countries

**DOI:** 10.1371/journal.pone.0157957

**Published:** 2016-06-30

**Authors:** Erwin De Cock, Persefoni Kritikou, Mariana Sandoval, Sunning Tao, Christof Wiesner, Angelo Michele Carella, Charles Ngoh, Tim Waterboer

**Affiliations:** 1 United Biosource Corporation, Barcelona, Spain; 2 United Biosource Corporation, London, United Kingdom; 3 United Biosource Corporation, Montreal, Canada; 4 Genentech Inc., South San Francisco, California, United States of America; 5 IRCSS AOU, San Martino, Italy; 6 F. Hoffmann-La Roche Ltd, Basel, Switzerland; Public Health England, UNITED KINGDOM

## Abstract

**Background:**

Rituximab is a standard treatment for non-Hodgkin lymphoma. The SABRINA trial (NCT01200758) showed that a subcutaneous (SC) rituximab formulation did not compromise efficacy or safety compared with intravenous (IV) infusion. We aimed to quantify active healthcare professional (HCP) time and patient chair time for rituximab SC and IV, including potential time savings.

**Methods:**

This non-interventional time and motion study was run in eight countries and 30 day oncology units. Rituximab SC data were collected alongside the MabCute trial (NCT01461928); IV data were collected per routine real-world practice. Trained observers recorded active HCP time for pre-specified tasks (stopwatch) and chair time (time of day). A random intercept model was used to analyze active HCP time (by task and for all tasks combined) in the treatment room and drug preparation area, drug administration duration, chair time and patient treatment room time by country and/or across countries. Active HCP and chair time were extrapolated to a patient’s first year of treatment (11 rituximab sessions).

**Results:**

Mean active HCP time was 35.0 and 23.7 minutes for IV and SC process, respectively (-32%, *p* <0.0001). By country, relative reduction in time was 27–58%. Absolute reduction in extrapolated active HCP time (first year of treatment) was 1.1–5.2 hours. Mean chair time was 262.1 minutes for IV, including 180.9 minutes infusion duration, vs. 67.3 minutes for SC, including 8.3 minutes SC injection administration (-74%, *p* <0.0001). By country, relative reduction was 53–91%. Absolute reduction in extrapolated chair time for the first year of treatment was 3.1–5.5 eight-hour days.

**Conclusions:**

Compared with rituximab IV, rituximab SC was associated with reduced chair time and active HCP time. The latter could be invested in other activities, whereas the former may lead to more available appointments, reducing waiting lists and increasing the efficiency of day oncology units.

**Trial Registration:**

ClinicalTrials.gov NCT01200758

## Background

Non-Hodgkin lymphoma (NHL) imposes a great burden on the healthcare system. It has been estimated that in Europe there were approximately 93,000 newly diagnosed cases and around 38,000 deaths in 2012 [1]. Over time, increased incidence and better survival rates for NHL led to greater demands on healthcare resources, putting increased pressure on capacity in oncology units [2].

NHL originates in the lymphatic system and can be divided into indolent (low-grade) and aggressive (high-grade) lymphoma. The most common types of low-grade and high-grade lymphoma are follicular (indolent) lymphoma (iNHL) and diffuse large B-cell lymphoma (DLBCL), respectively [3].

Rituximab (MabThera^®^; Rituxan^®^) [4] is a chimeric monoclonal antibody targeting the CD20 antigen which is present on the surface of B-lymphocytes and is characterized as a relatively non-toxic drug that can be combined with chemotherapy regimens [5]. Rituximab-containing regimens have become a standard treatment for iNHL and DLBCL [6] with a standard dose of 375 mg/m^2^ body surface area administered by intravenous (IV) infusion [7]. In iNHL, it is used in combination with chemotherapy in patients with advanced disease who have not been treated before, or as maintenance therapy in patients whose follicular lymphoma has responded to initial chemotherapy. It is also used as monotherapy in patients with advanced disease, who are resistant to chemotherapy or who have failed two or more chemotherapy treatments. In DLBCL, rituximab is used in combination with cyclophosphamide, doxorubicin, vincristine and prednisolone (CHOP) [4].

When administered via IV infusion, infusions of rituximab typically last for three to four hours [8]. Hence, administration of rituximab IV can be considered resource intensive for the healthcare system and time consuming for patients.

A subcutaneous (SC) formulation of rituximab, administered via hand-held syringe (HHS), obtained European Medicines Agency approval as an alternative to the IV infusion in both iNHL and DLBCL. Rituximab SC contains recombinant human hyaluronidase (rHuPH20), an enzyme used to increase the dispersion and absorption of co-administered substances when administered subcutaneously. Approval was based on evidence from two studies. The Phase Ib Sparkthera trial (BP22333; NCT00930514) demonstrated non-inferior pharmacokinetics and safety of rituximab SC compared with rituximab IV administration as maintenance therapy in patients with iNHL [9, 10]. The stage 1 results of the Phase III SABRINA trial (BO22334; NCT01200758) in follicular lymphoma showed that the pharmacokinetic profile of rituximab SC was non-inferior to rituximab IV and was not associated with new safety concerns [11]. Before starting rituximab SC, all patients must always receive a full dose of rituximab IV beforehand, followed by up to seven subsequent cycles with rituximab SC injected at a fixed dose of 1,400 mg per cycle during the induction phase and once every two months as a maintenance treatment until disease progression or for a maximum period of two years [8].

The Sparkthera trial also showed that SC injection can be administered in approximately five minutes [9,10]. Similarly, the SABRINA study showed that rituximab SC shortened administration time to approximately five to seven minutes [11].

It is expected that a transition from rituximab IV to SC would reduce patient chair time, active healthcare professional (HCP) time and consumables [12]. A recent study comparing IV and SC formulations of another monoclonal antibody, trastuzumab, showed that active HCP time was reduced by a mean of 13 minutes per session with the SC single-use injection device (SID) and 17 minutes with the SC HHS. Patient chair time was reduced by 55 and 57 minutes, respectively [13]. Two studies in the UK showed that substituting trastuzumab IV and rituximab IV with trastuzumab SC and rituximab SC, respectively, was associated with reduced active HCP time, patient time and costs [14,15]. Both UK studies used the same study protocol as the global time and motion studies for trastuzumab and the study reported here, but applied a definition of active HCP time whereby total IV infusion duration was considered active time attributable to a single patient.

This time and motion study was designed to quantify active HCP time as well as chair time associated with rituximab IV and SC administrations in patients with iNHL. A secondary objective was to estimate the potential time savings with a conversion from rituximab IV to SC, for a single administration session and for the first year of treatment. Rituximab SC observations were collected on patients participating in the Phase IIIb MabCute trial (MO25455; NCT01461928: 87% of centers were public and 13% of centers were private), a randomized parallel-group trial designed to evaluate the efficacy and safety of maintenance therapy with rituximab SC until disease progression in patients with relapsed or refractory iNHL who responded to rituximab-based immunotherapy induction and initial two-year rituximab maintenance therapy administered subcutaneously [16].

## Methods

### Study Design

This was a multinational, multicenter, non-interventional, time and motion study conducted in eight countries and 30 day oncology units. Time and motion methodology was used to identify all relevant steps in both rituximab SC and IV processes and to collect time actively spent by HCPs on pre-selected tasks as well as chair time. Key study definitions are presented in Table 1.

**Table 1:** Study Definitions.

Term	Definition
**Active HCP Time**	Time actively dedicated by any staff member on pre-specified tasks.
**Observed Time**	Time collected through stopwatch measurement for pre-specified tasks related to the rituximab preparation (IV reconstitution or SC syringe filling), dispensing, treatment administration and post-treatment monitoring.
**Chair Time**	Time between entry and exit of patient chair.*
**Treatment Room**	The place where IV and SC rituximab treatments are being administered.
**Patient Treatment Room Time**	Time between entry in treatment room for receiving rituximab therapy and exit of treatment room.
**DPA**	The place where IV rituximab reconstitution and SC hand-held syringe filling is typically performed, prior to the actual treatment administration. The DPA can refer to the hospital pharmacy or to a special aseptic DPA within the day oncology unit.

*In the case of induction therapy, with same-day concomitant infusion(s), exit time was adjusted to reflect the time when activities related to rituximab were completed.

HCP: healthcare professional; IV: intravenous; SC: subcutaneous; DPA: drug preparation area.

For rituximab SC, data were collected for both induction therapy (typically followed by same day administration of chemotherapy) as well as maintenance therapy (single rituximab SC injection) in the relapsed/refractory iNHL population in the MabCute trial. At the same centers, real-world rituximab IV processes were observed during the same data collection period (May 2012 to December 2013) in patients with either relapsed/refractory iNHL or previously untreated iNHL. The term “real-world” refers to processes performed in routine clinical practice, as opposed to those happening within a clinical trial setting. Rituximab IV was administered as monotherapy to avoid any potential impact on process flow due to chemotherapy agents being administered concomitantly. The exception was Brazil where patients received IV rituximab together with chemotherapy on the same day, as per usual clinical practice. Although rituximab SC process was governed by trial protocol, limited variation in time for observed tasks was expected with future real-world application. Hence, time and motion data collected per protocol were expected to be a good proxy for data observed in the real world.

### Regulatory and Pharmacovigilance Procedures

A separate time and motion sub-study protocol was prepared and submitted to the appropriate national competent authorities in charge of reviewing the MabCute study protocol and wherever required, additional submissions were made to local ethics committees.

The following ethics committees, listed by country, considered and approved the time and motion sub-study protocol:

**Austria:** Ethikkommission der Medizinischen Universität Graz; **Brazil:** Comitê de Ética em Pesquisa do Instituto de Saúde e Bem-estar da Mulher; Comitê de Ética em Pesquisa da Casa de Saúde Santa Marcelina; CEP da Fundacao Pio XII—Hospital de Cancer de Barretos; **France:** Comité de Protection des Personnes "Est-I", Faculté de Médecine, BP 87900, 21079 Dijon Cedex; **Italy:** Comitato Etico per la Sperimentazione Dell`azienda Ospedaliera di Padova, Comitato Etico dell`Azienda Ospedaliera Universitaria S. Martino di Genova; Comitato Etico dell`Azienda Ospedaliera Antonio Cardarelli di Napoli, Comitato Etico dell'Azienda Policlinico Umberto I di Roma; **Russia:** Ethics Committee of FSBI "Russian Oncology Research Center named after N.N. Blokhin" of RAMS; **Slovenia:** Komisija Republike Slovenije za Medicinsko Etiko; **Spain:** CEIC Hospital Universitario Vall D´Hebrón; **UK:** National Research Ethics Service RES- Committee East England, Cambridge South.

Considering that rituximab SC was an investigational treatment, patients provided informed consent and were treated according to the MabCute study protocol. No informed consent was required for patients receiving rituximab IV, as only HCPs (and not patients) were observed. For SC observations, solicited safety reporting was specified in the study protocol of the MabCute trial.

### Data Collection

**Fig 1:**
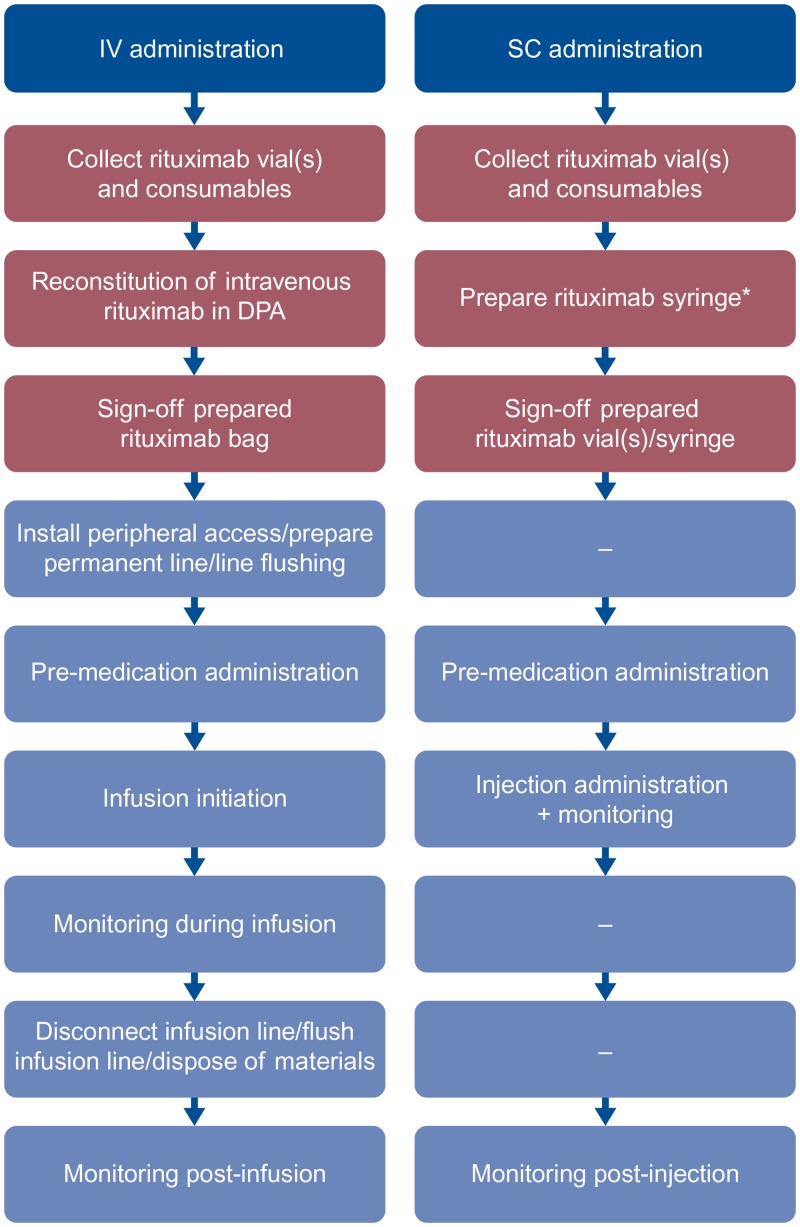
Chronological listing of tasks measured. DPA tasks are in red and treatment room tasks are in blue. *Rituximab syringe can be filled either in the pharmacy (43%), or in a special aseptic DPA within the day oncology unit (57%). IV, intravenous; SC, subcutaneous; DPA, drug preparation area.

Three generic case report forms (CRFs) were developed listing, in chronological order, pre-selected tasks that constituted the IV and SC processes in the treatment room and the IV and SC preparation and dispensing processes in the drug preparation area (DPA) (Fig 1 and S1–S3 Tables). Special attention was given to ensure that all tasks that were deemed potentially different between IV and SC were included. Tasks expected to be equivalent (i.e., patient arrival/registration, blood sampling and physician consultation visit) were excluded.

Following interviews with a nurse and pharmacy member in each center, the generic CRFs were customized, if needed, with updated task descriptions reflecting local center practice to ensure accurate time measurements.

Two types of time measurement were performed: “stopwatch” time (minutes and seconds) to measure active HCP time and “time of day” (hours and minutes) to measure infusion duration and chair time. Other data collected included: first or subsequent infusion of rituximab IV; induction or maintenance treatment of rituximab SC; presence of a permanent infusion line (and type of permanent line) or need for a peripheral catheter; need for premedication drugs (including route of administration and dose); for each task, the type of HCP performing the task (e.g., registered nurse); detail on potential unexpected activities that were not related to the patient or the task being observed. No patient demographic data or clinical measures were collected.

At least two observers per center were trained and observations were performed randomly between observers. Data collection was monitored remotely and data queries were clarified by observers on data clarification forms.

### Statistical Analyses

This was a descriptive (non-hypothesis-testing) study. The number of centers and sample sizes were based on convenience and feasibility.

Each completed CRF represented an observation. Multiple observations were allowed for the same patient. Observations performed in the treatment room and in the DPA were independent, resulting in different sample sizes by setting (Table 2).

**Table 2:** Number of Completed Observations by Country.

		IV Process		SC Process	
Country	Centers (n)	Treatment Room	DPA	Treatment Room	DPA
Austria*	4	76	75	16	15
**Brazil**	3	75	74	76	76
**France**	6	65	52	50	41
**Italy**	4	47	52	43	23**
**Russia**	6	121	121	60	60
**Slovenia**	1	26	26	6	5
**Spain**	3	90	108	34	34
**UK**	3	38	13**	30	20**
**Total**	30	538	521	315	274

*In Austria, one center contributed only one IV observation for the treatment room and two centers did not contribute any SC observations.

** The DPA observations in the UK and Italy were for multiple patients.

IV: intravenous; SC: subcutaneous, DPA: drug preparation area.

In case task time was not reported, a zero value was entered when it was ascertained that the task did not occur. A data point was considered missing when it was unclear whether the task occurred or not. Imputation (using the mean estimate of available observations) was done if a task consisted of two or more sub-tasks and absence of observed time for one or more subtasks would underestimate total task time. Per task and per center, outlier values were consistently queried as part of a formal data clarification process and a value could be excluded from the analysis if the center could not certify its correctness.

Data were analyzed using SAS 9.1 (SAS Institute Inc., Cary, North Carolina, USA), either by country and/or for all countries combined. Two analytical approaches were applied to analyse active HCP time: a task-based and a case-based approach.

#### Per-Country Analyses

In the treatment room, each pre-specified task was treated as an independent data sample (task-based approach). A random intercept generalized linear mixed effects model (hereafter, random intercept model) tested whether time was clustered by center. If a statistically significant center effect was detected at α = 0.05, adjusted mean time and its corresponding confidence interval (CI) was used; otherwise, the mean and corresponding CIs were calculated based on the best fitting distribution (based on goodness-of-fit-testing). To calculate total active HCP time in the treatment room for each process, the mean times from each task were summed up to a composite mean total time. Active HCP time in the DPA (single time variable), infusion duration, chair time and patient treatment room time were analyzed using the same process as described above.

#### Pooled Countries Analyses

For each observation or case, active HCP time in the treatment room was calculated as the sum of the time for each task. If time for a task was missing, average time across all other cases in that center was imputed (otherwise, total active HCP time for that case would be underestimated). The composite total active HCP time variable was analyzed for all countries combined using the random intercept model, generating an adjusted mean and corresponding CI. Pooled countries analyses were also conducted on: active HCP time in DPA, injection time, infusion duration, chair time and patient treatment room time, using the same random intercept model.

#### Post Hoc Exploratory Analyses

A post hoc analysis on the pooled dataset explored potentially significant differences in active HCP time in the treatment room, active HCP time in the DPA and chair time between rituximab IV and SC.

### Extrapolating Active HCP Time and Chair Time for First Year of Treatment

Active HCP time and chair time per session were extrapolated to a patient’s first year of rituximab treatment assuming 11 administrations (eight induction and three maintenance sessions). As part of the country analyses, distributions of active HCP time by HCP type were calculated for each country and extrapolated results are shown by level of HCP involvement.

To obtain a more accurate estimate of extrapolated chair time, different time estimates for first and subsequent infusion were used. In countries with five or more first infusion observations (cut-off value based on sample sizes of first infusions by country), extrapolated chair time was calculated as the mean time for a first infusion plus 10 times the mean time for a subsequent infusion. If there were less than five first infusion observations, duration of a first infusion was estimated as the mean for a subsequent infusion plus the mean difference in chair time between first and subsequent infusion as obtained from the pooled countries analysis. In the case of rituximab SC, the first session was assumed to be an IV infusion (the 10 remaining sessions were SC).

## Results

### Number of Observations

Table 2 summarizes the participating countries and number of observations by country. Across all rituximab IV observations, 43 (8%) were first infusions and 495 (92%) were subsequent infusions. In the rituximab SC sample, 217 (69%) were induction treatment and 98 (31%) were maintenance treatment observations.

### Active HCP Time

**Fig 2:**
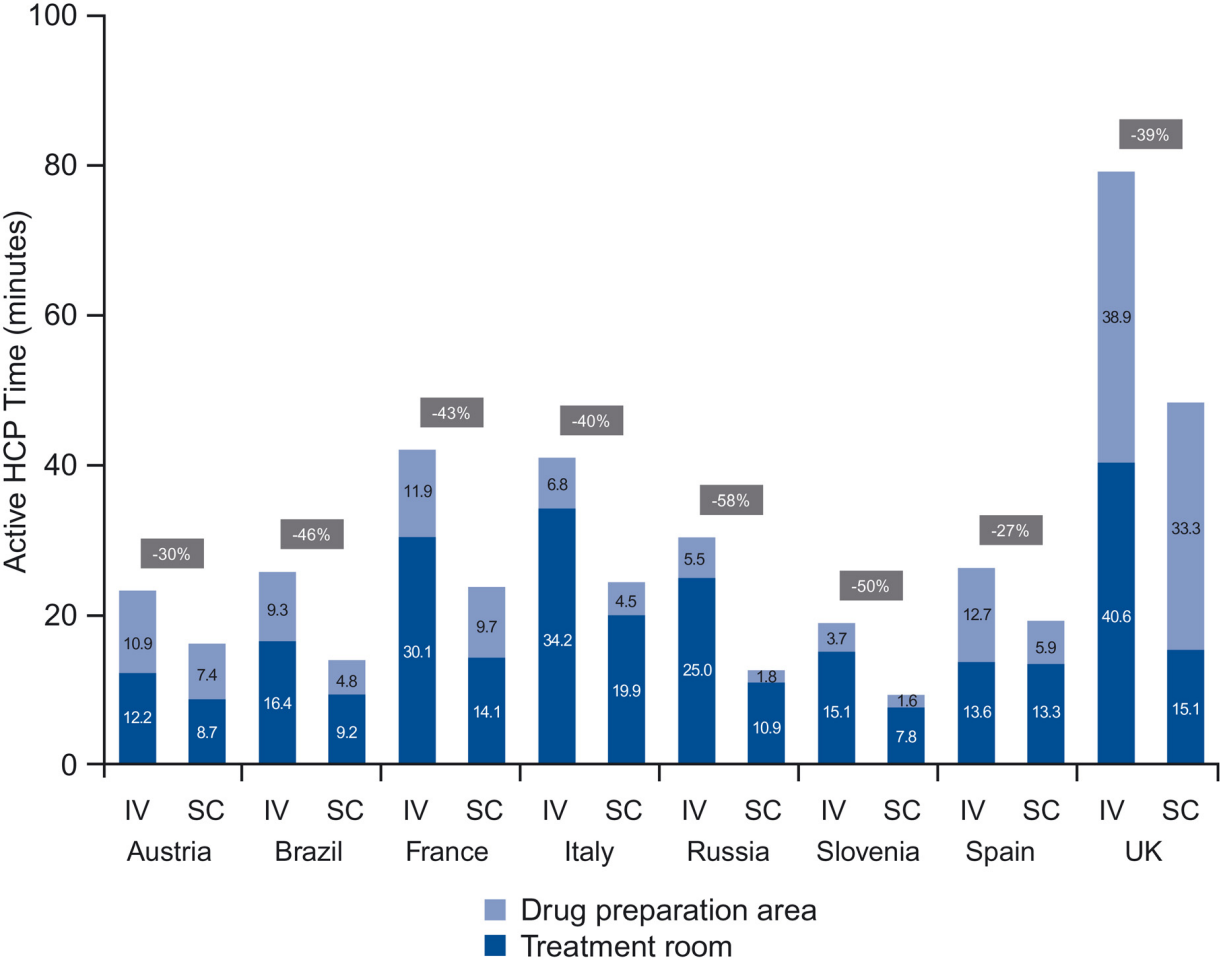
Active HCP time in treatment room and DPA (per session). HCP, health care professional; DPA, drug preparation area; IV, intravenous; SC, subcutaneous.

When pooled, mean IV process time was 35.0 minutes (95% CI 29.7–40.3) compared with mean SC process time of 23.7 minutes (95% CI 18.7–28.8), or a significant reduction of 11.3 minutes (-32%) across the treatment room and DPA combined *(p <*0.0001) (based on the random intercept model). For IV, mean time spent on treatment room tasks ranged from 12.2 minutes (Austria) to 40.6 minutes (UK) and for SC from 7.8 minutes (Slovenia) to 19.9 minutes (Italy). DPA task time for IV ranged from 3.7 minutes (Slovenia) to 38.9 minutes (UK) and from 1.6 minutes (Slovenia) to 33.3 minutes (UK) for SC. The share of DPA time in total active HCP time ranged between 17% (Italy) and 49% (UK) for IV and between 14% (Russia) and 69% (UK) for SC (Fig 2).

**Fig 3:**
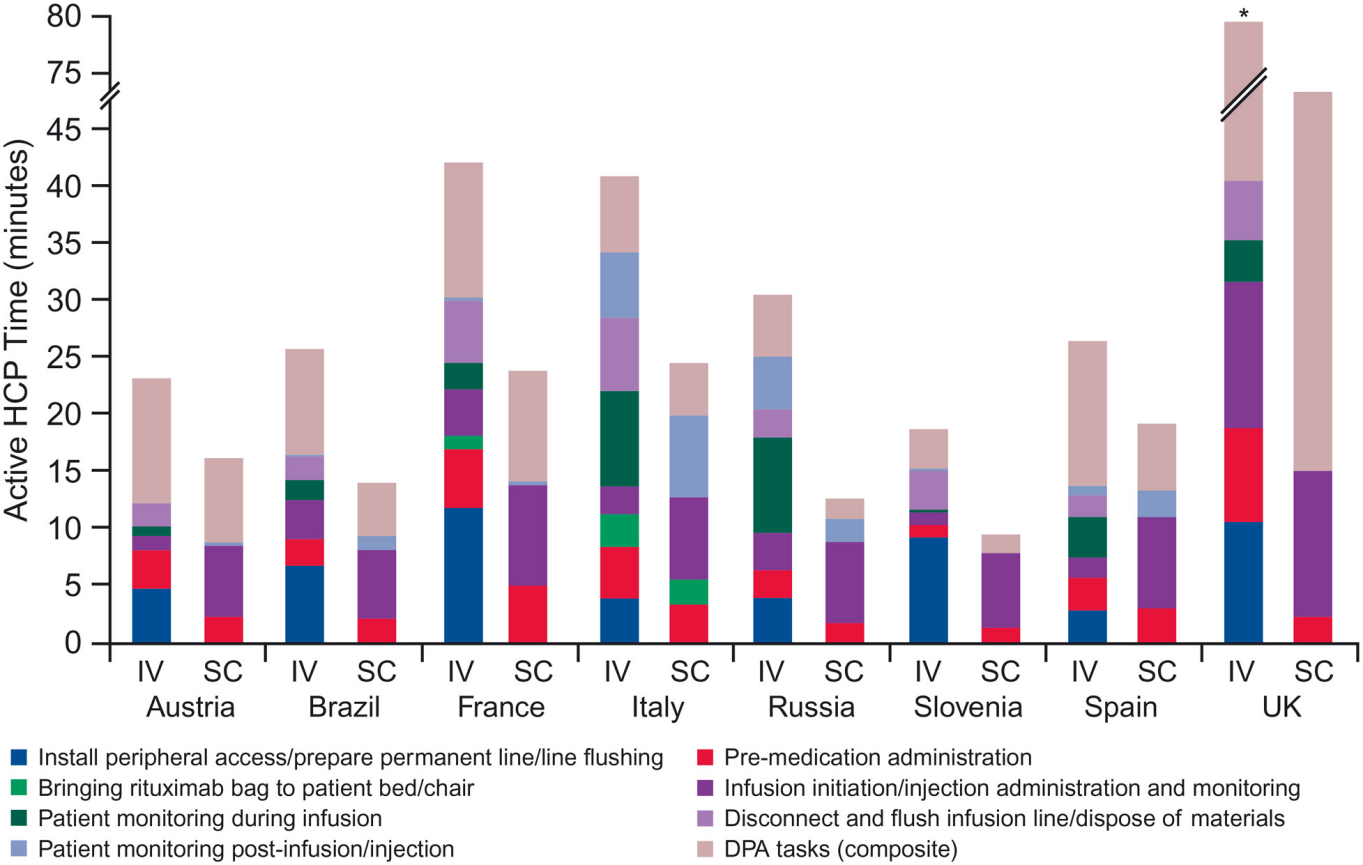
Active HCP time in treatment room and DPA, by task (per session). The mean country estimate for each task is provided in S4 Table. Total active HCP time for IV administration in the UK was 79.5 minutes. HCP, healthcare professional; DPA, drug preparation area; IV, intravenous; SC, subcutaneous.

Important differences in task composition and absolute times were observed within countries (not shown) and between countries for both IV and SC processes (Fig 3). This was due to the observational nature of the study. Countries and also centers within a country may have different routine clinical practices leading to different process flows and consequently different time requirements. Use of different observers and the observation of different staff performing the tasks may also have contributed to the observed variability. Some reasons for between-country variability, due to differences in process flows, are described in the discussion.

For IV, the main contributors of active HCP time in the treatment room were: “install venous catheter/line flushing” in Austria (4.7 minutes), Brazil (6.7 minutes), France (11.7 minutes) and Slovenia (9.1 minutes); “infusion initiation” in the UK (12.8 minutes); and “active monitoring during infusion” in Italy (8.3 minutes), Russia (8.4 minutes) and Spain (3.5 minutes). For SC, the main driver was “injection administration” ranging from 6.0 minutes (Brazil) to 12.9 minutes (UK). S4 Table indicates which results were derived from the random intercept model vs. those that came from the standard regression.

When comparing total active HCP time for both processes, reductions ranged from 27% (Spain) to 58% (Russia). Reductions in the treatment room ranged between 2% (Spain) and 63% (UK) and in the DPA ranged between 14% (UK) and 67% (Russia). Principal reasons for reduced time with SC were fewer DPA activities and not having to install and disconnect a peripheral catheter (or flush a permanent line). However, savings were partially offset by increased SC injection time (compared with infusion initiation).

**Fig 4:**
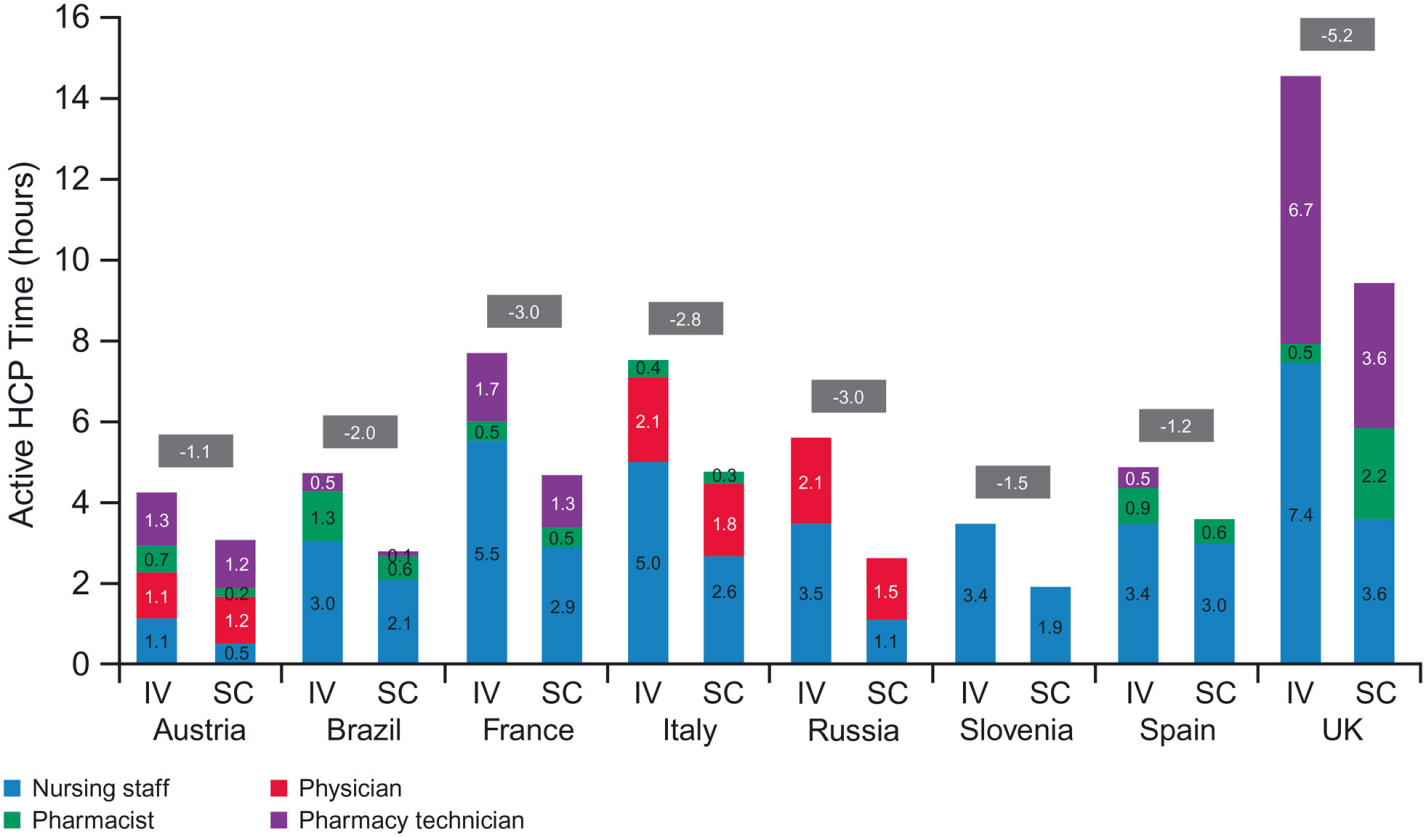
Active HCP time in treatment room and DPA, by HCP type (11 sessions). HCP, healthcare professional; DPA, drug preparation area; IV, intravenous; SC, subcutaneous.

Fig 4 shows extrapolated active HCP time for the first year of treatment. Estimated savings with a switch from IV to SC ranged between 1.1 hours (Austria) and 5.2 hours (UK). Fifteen different HCP types were grouped into four categories: nursing staff, physician, pharmacist and pharmacy technician/assistant staff. Nursing staff played the biggest role in both processes across most countries (exceptions were Austria and Russia). The physician had a high level of involvement in Russia, Italy and Austria, especially in the SC process (63%, 40% and 40%, respectively), while pharmacy technician staff were important in Austria (31% for IV and 41% for SC) and the UK (46% for IV and 37% for SC).

### Patient Time

The pooled countries analysis showed mean IV chair time of 262.1 minutes (95% CI 228.4–295.7) compared with a mean time of 67.3 minutes for SC (95% CI 43.1–91.5), or a 74% reduction (*p* <0.0001). Results were driven by IV infusion duration (180.9 minutes [95% CI 153.7–208.1]) compared with SC injection time (8.3 minutes [95% CI 7.0–9.6]). Consequently, patients receiving rituximab IV spent more time in the treatment room, with a mean of 281.8 minutes (95% CI: 246.9–316.7) vs. 95.9 minutes (95% CI: 68.1–123.7) for rituximab SC. All results were derived from the random intercept model (center effects were detected).

**Fig 5:**
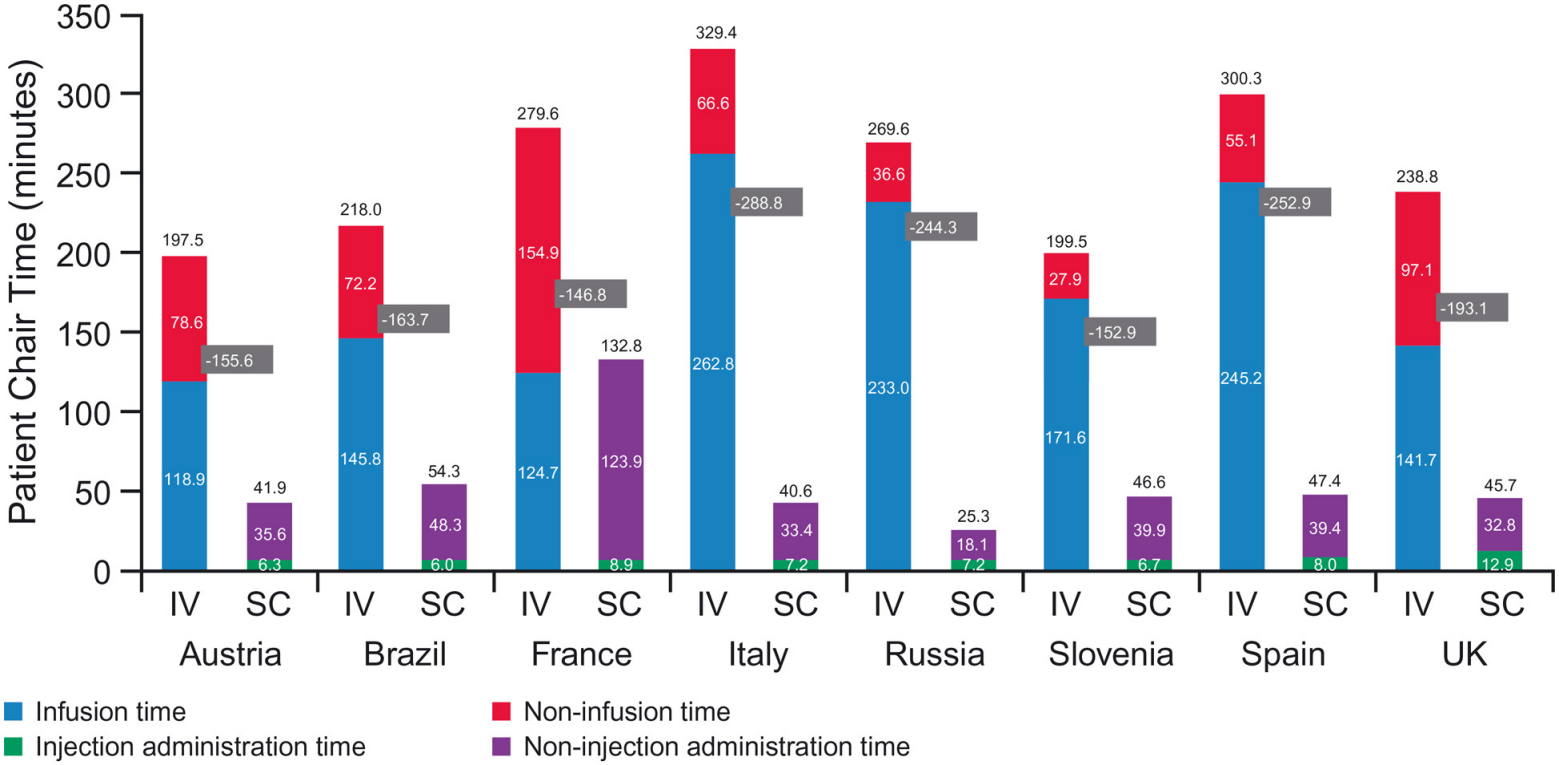
Patient chair time for IV vs. SC administration (per session). Results derived from the random intercept model: infusion time (France, Russia), injection administration time (France), IV patient chair time (France), SC patient chair time (France). All other results were derived from the standard regression analysis. IV, intravenous; SC, subcutaneous.

By country, relative reduction in mean chair time for SC (compared to IV) ranged between 53% (France) and 91% (Russia), while absolute reduction ranged between 146.8 minutes (France) and 288.8 minutes (Italy) (Fig 5). For infusion duration, absolute reduction in time ranged from 118.9 minutes (Austria) to 262.8 minutes (Italy), or a relative reduction of 91% (UK) to 97% (Italy, Russia and Spain) (Fig 5).

**Fig 6:**
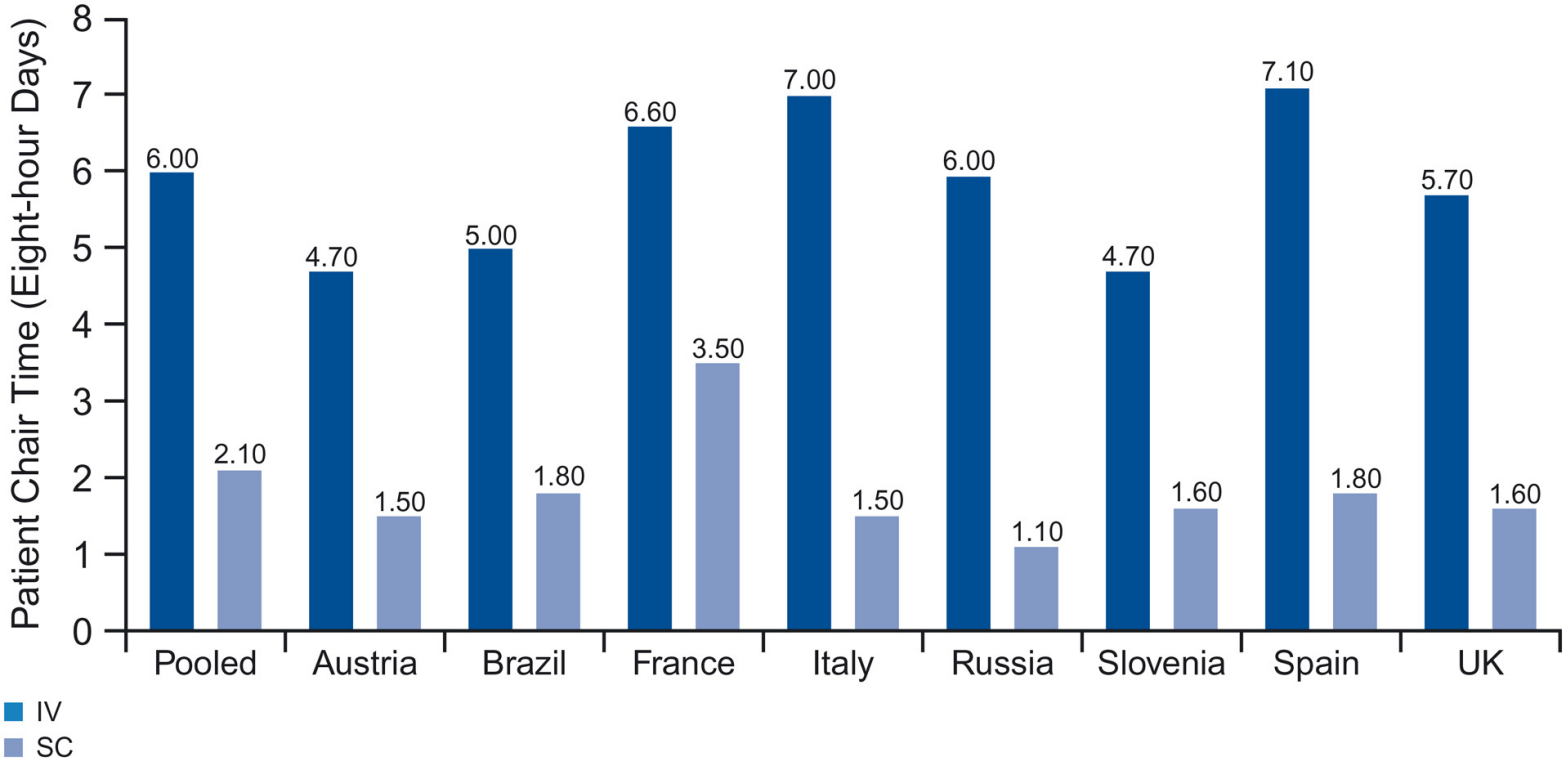
Patient chair time for first year of treatment (11 sessions).

The pooled countries analysis confirmed a statistically significant increase in infusion duration and consequently chair time for “first versus subsequent infusion” with a mean increase in chair time for a first infusion of 83.1 minutes (*p* <0.001) (Fig 6).

**Fig 7:**
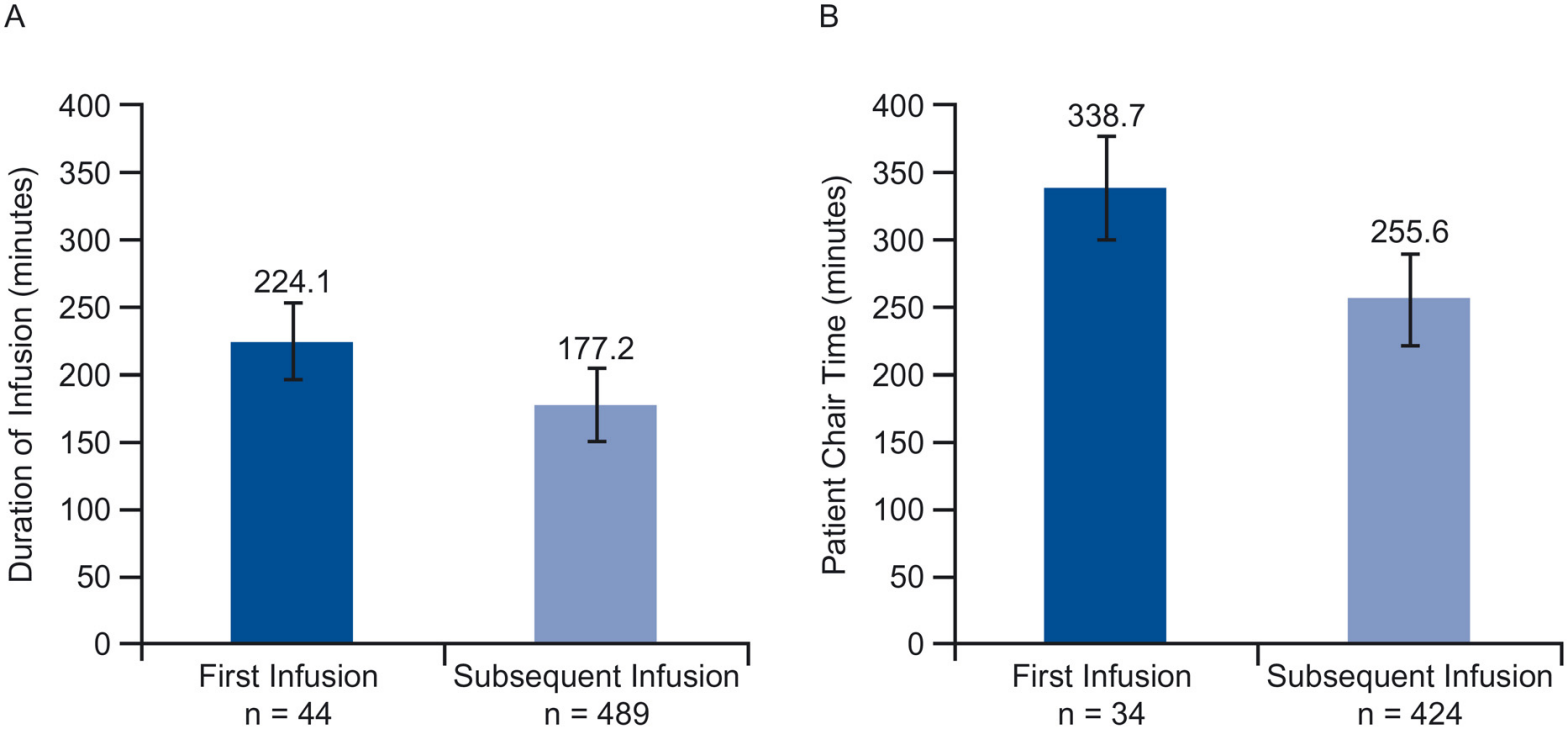
Impact of first vs. subsequent infusion on (A) infusion duration and (B) patient chair time.

Using those results yielded 3.9 eight-hour days of chair time freed up annually per patient treated with SC (first infusion still being IV). By country, absolute reduction in extrapolated chair time for the first year of treatment ranged between 3.1 (France) and 5.5 (Italy) eight-hour days; relative reduction was between 47% (France) and 82% (Russia) (Fig 7).

## Discussion

This time and motion study revealed that rituximab SC was associated with important reductions in chair time and active HCP time compared with rituximab IV. When all countries were pooled, a statistically significant reduction in active HCP time across both the treatment room and the DPA of 11.3 minutes (-32%) was shown. Most of this reduction (92%) was attributable to treatment room tasks, in particular to the fact that SC process does not require the installation and disconnection of a peripheral catheter (or the flushing of a permanent infusion line), or monitoring time associated with the infusion. When extrapolating active HCP time to a patient’s first year of rituximab treatment (eight induction plus three maintenance sessions) by country, this time reduction translated into 1.1 to 5.2 hours of active HCP time saved annually, mainly as a result of reduced nursing staff involvement.

Activities that were expected to be similar between IV and SC processes (such as blood sampling, physician consultation visits, patient registration and administrative tasks) were not included in the study. For those tasks that were included, task descriptions and the workload distribution by HCP types varied across centers and countries. For example, “bringing rituximab bag to patient bed/chair” was either a separate step, or combined with “infusion initiation”. “Install venous catheter/line flushing” depended on the proportion of patients needing a peripheral catheter (as opposed to a permanent line already being installed). For SC, the syringe could be filled in the hospital pharmacy or in a special aseptic DPA within the day oncology unit prior to injection.

Observed differences in process flows led to different active HCP time estimates and differences in the potential for time savings with a switch to SC formulation (as shown in Fig 3). For example, the Spanish centers appeared to be very efficient with the IV process in the treatment room, with almost equal time for IV (13.6 minutes) and SC (13.3 minutes). Across the Russian centers, the higher relative reduction in treatment room time was due to a mean 8.4 minutes active monitoring during infusion (estimates in other countries ranged between 0.9 and 3.7 minutes, except in Italy with 8.3 minutes). In the UK, sites explained that high pharmacy preparation time was due to established protocols for IV and longer time for quality control and checking times for SC (due to the MabCute trial).

Pooled countries analyses showed a statistically significant reduction in chair time of 194.8 minutes, which was driven by reduced time for rituximab administration (180.9 minutes for IV vs. 8.3 for SC). Any IV monotherapy process was eligible for observation, including rapid infusion, according to each center’s routine practice. It should be noted that the discrepancy between chair time and administration time is because of substantial chair time reported prior to and post-drug administration. By country, the estimated annual chair time saved per patient ranged between 3.1 and 5.5 eight-hour days (average 3.9). Taking the average of all countries, 3.5 patients could be treated with SC (first session IV and 10 sessions SC) for every patient treated with IV. This shows the potential for increased capacity and a higher number of available day oncology unit appointments, therefore allowing waiting lists to be cut. Though it would be interesting to calculate the total cost per patient for the administration of rituximab IV and SC (including staff time, drug and infusion chair resources), this was outside the remit of the present study. Such analysis would require different perspectives: the hospital that pays for those resources and the health system that may fund an administration session on a fee-for-service basis. Also, costs within multinational studies are not easily comparable due to different HCP salary structures and country costing levels. Contrary to drug and consumable usage, which have a direct measurable impact on cost, “active” HCP time and chair time savings would only translate in true cost savings if staff and infusion chair capacity were reduced, which is not realistic in the short term. Savings in the opportunity cost for infusion chair capacity (i.e., the value of the best available use of the chair) are expected to be far greater than those resulting from “active” HCP time. For illustrative purposes, an hourly infusion chair cost of €56 was assumed, based on analytical accounting data from one center in Switzerland. This included depreciation of equipment, utility costs required to run the infusion suite, staff support functions and other overhead costs (Dr. Nik Hauser, personal communication 2014). The reduction in chair time (from pooled countries analysis) translated into annual opportunity cost savings of €1,761 per patient. Future research is warranted in order to estimate the full cost implications of a switch from rituximab IV to SC both at the hospital and the health system level.

From a societal perspective, there are benefits for the patients and their caregivers. Indeed, reduced chair time would translate into reduced time in the hospital (assuming all other hospital-based activities for both IV and SC processes are equal). There is also the potential benefit of convenience [17]. In a breast cancer population, the PrefHer study (NCT01401166) which was designed to assess patients’ preferences for the trastuzumab SC SID or SC HHS over IV infusion showed that the majority of patients preferred SC regardless of the administration method, primarily because SC saved them time [18]. A similar study, PrefMab (NCT01724021), presented similar results in terms of patients’ preferences for rituximab SC over rituximab IV, with the main reasons being the reduced patient time in the clinic, the reduced emotional distress and the more comfortable administration [19].

In order to account for potential clustering of data by center, due to aforementioned differences in center practice flows, the per-country analyses employed a random intercept model and, if no effect was detected, used the best fitting distribution. In both cases, large CIs (not shown) indicated a lack of sufficient information for an informative generalization at the respective country level (i.e., the true country mean can fall anywhere within the CI). When pooling all centers across all countries (30 centers provided a much larger sample), the confidence intervals for both IV and SC processes became narrower and further exploratory analyses were able to show true differences between both routes of administration.

A key strength of the present study was the robustness in applying the concept of active HCP time. Previous studies in the UK [14,15] assigned total IV infusion duration as active time dedicated to a single patient. However, it is reasonable to assume that a nurse cares for multiple patients simultaneously or performs non-patient-care activities during infusion. The estimates of active HCP time presented here are likely conservative as it could be argued that some but not all of the “non-active” time may be attributed to a single patient.

### Limitations

The present descriptive study was limited by its non-comparative, hypothesis-generating design. In the absence of a priori evidence to inform expected mean active HCP times and variability for both processes, including evidence on within center and between center variance, a sample size calculation was not performed. Also, no real-world experience for rituximab SC was available at the time of study set up. This was less of an issue for chair time because difference in IV infusion duration (two to four hours based on rituximab IV label) and injection duration from earlier clinical studies (SABRINA and SparkThera estimated five to seven minutes) was expected to be substantial. Additionally, SC sample sizes were conditioned by the MabCute trial and IV sample sizes by the number of patients receiving IV rituximab in each center. As the study centers enrolled different numbers of patients and were activated at different time points during the trial, there was an imbalance in the number of observations between study groups and between centers.

SC observations were collected as part of the MabCute trial, whilst IV observations were performed in a real-world setting. Both settings may be associated with slightly different treatment practices. For example, it is often the case in a clinical study setting that administrative duties for drug dispensing in the DPA are performed by more senior staff. This was observed in the UK, where mean pharmacy technician time for rituximab IV was shifted to pharmacist time for rituximab SC, resulting in somewhat higher drug preparation costs for rituximab SC, as a result of higher pharmacist salary [14]. It could reasonably be expected that drug preparation time would be lower if SC rituximab were in wider use. In the treatment room, rituximab SC patients were treated within the same observation unit environment and by the same staff as they would be if they were not taking part in a clinical study.

Ideally only monotherapy observations for SC and IV rituximab would have been observed to provide a clean comparison of the two formulations. For rituximab SC, maintenance observations only (single SC injection of rituximab) would have prevented data collection during the first six to eight months of the trial. Hence, data were also collected for induction therapy (followed by same day administration of chemotherapy), which made up 69% of all SC observations. For those observations, patient time variables were adjusted to reflect the time associated with rituximab only. Indeed, patients on induction therapy receive a variety of chemotherapy regimens and therefore the variation in patient chair/treatment room/care unit time would be large. For IV, monotherapy processes were observed; except for five induction observations in the UK (patient time variables were also adjusted). In this way, all patient time data were made comparable across both groups. With regards to active HCP time, we expect that the inclusion of induction observations in the SC sample may have increased the average time for the SC process because of the possible addition of “installation of peripheral catheter/flushing of permanent line”, either prior to or post-SC rituximab injection. In most cases, a permanent line was already installed. Therefore, when exploring the impact of a switch from IV to SC, our analysis is conservative because adding additional time reduces the potential to show time savings. Because of the nature of observational studies and measurement of time outcomes in particular, variability in task time is inevitable and the result of various factors mutually interacting: center, process characteristics (e.g., first vs. subsequent infusion), patient characteristics (e.g., severity and co-morbidities), the observers who measured the time and the HCPs being observed. Especially “center” was expected to have an impact on time, as was shown by the random intercept model on the pooled datasets. The impact on time of other aforementioned variables (i.e., process characteristics, patient characteristic, observers and HCPs) is more difficult to analyze and therefore less clear. As part of exploratory covariate analyses within each country, the impact of some process characteristics (“first versus subsequent infusion”, “peripheral access versus permanent line”) on active HCP time, infusion duration and chair time was studied. However, no impact could be detected, probably due to insufficient sample sizes. Within the pooled sample, a statistically significant difference in infusion duration and chair time was detected between first and subsequent infusion (in line with expectation based on the rituximab IV label). No patient characteristics, including demographics or clinical information, were collected and hence this study was not able to evaluate the association between time and such characteristics. The potential impact of inter-observer variability on stopwatch measurement was mitigated firstly by agreeing on task descriptions with clear start and stop points and secondly by performing standardized training. Also, the same observers were used to monitor active HCP time for SC and IV rituximab processes within each center. Lastly, bias could have been introduced into the study by inevitable variation in the time individual HCPs take to perform tasks and due to the Hawthorne effect (i.e., potential change in HCP behavior because of being directly observed) [20]. This was mitigated by clearly informing all study staff of the study aims and that study focus was not on individual staff efficiency measurement, but overall process efficiencies instead.

## Conclusions

For the main study outcomes of active HCP time in treatment room, active HCP time in DPA and chair time, post hoc analysis on the pooled dataset across all centers showed statistically significant reductions between IV and SC (*p* <0.0001). This study also showed important variability in absolute times and time reductions between countries, as a result of differences in center process pathways. Active HCP time saved could be invested in other patient care activities. A reduction in chair time may lead to a higher number of available day oncology unit appointments and therefore allowing waiting lists to be reduced. Indeed, across all countries, an average 3.5 patients could be treated with SC (first session IV and 10 sessions SC) for every patient treated with IV, or time freed up could be used to administer other treatments. The financial implication could be a substantial reduction in costs related to infusion chair equipment (assuming that infusion chairs are variable and not needed in the long run), or the opportunity to generate other revenue in capacity-constrained centers. Rituximab SC administration therefore offers the potential to enhance the efficiency of oncology units and improve convenience for patients.

## Supporting Information

S1 Table Rituximab IV Generic Observation Form. IM, intramuscular; IV, intravenous; SC, subcutaneous; NaCl, sodium chloride.
(DOCX)

S2 Table Pharmacy Generic Observation Form. IV, intravenous.
(DOCX)

S3 Table Rituximab SC Generic Observation Form. IM, intramuscular; IV, intravenous; SC, subcutaneous.
(DOCX)

S4 Table Active HCP Time in Treatment Room and DPA, by Task and by Country (per session). DPA: drug preparation area; HCP: healthcare professional; IV: intravenous; SC: subcutaneous. *Indicates results that were derived from the random intercept model; all other results were derived from the standard regression analysis (gamma was the best fitting distribution in almost all cases).
(DOCX)
