# Supplementary material for: Time Savings with Rituximab Subcutaneous Injection versus Rituximab Intravenous Infusion: A Time and Motion Study in Eight Countries
Source: PLoS One. 2016 Jun 30;11(6):e0157957. doi: 10.1371/journal.pone.0157957 (PMC4928781; doi:10.1371/journal.pone.0157957)
Supplement: S1 Table — IM, intramuscular; IV, intravenous; SC, subcutaneous; NaCl, sodium chloride. (DOCX) [file pone.0157957.s001.docx]

**S1 Table. Rituximab IV Generic Observation Form.**

| **Install Peripheral Catheter/Line Flushing** | |
| --- | --- |
| START: | Go to patient bed/chair. |
| *[if permanent line]* | Connect infusion line and prepare infusion pump. |
| *[if peripheral access]* | Insert peripheral catheter, connect infusion line and prepare infusion pump. |
| STOP: | Leave patient bed/chair. |
| **Pre-medication Administration** | |
| If pre-medications are conducted as a single activity | |
| START: | Go to preparation area and collect all required pre-medications. Go to patient bed/chair. |
| *[oral drug]* | Medication and something to drink given to patient and uptake is controlled. |
| *[IV drug]* | Remove pre-medication from packaging; administer the medication via bolus IV infusion. |
| *[IM or SC drug]* | Remove pre-medication from packaging; administer the medication via direct injection. |
| *[if required]* | Administer further pre-medications. |
| *[after final pre-medication administration]* | Flush line with NaCl solution via bolus injection. |
| STOP: | Leave patient bed/chair OR return to preparation area. |
| If pre-medication administration is conducted as a sequence of separate activities | |
| START: | Go to preparation area. |
| Remove first pre-medication from packaging, go to patient bed/chair and connect medication to patient IV line. | |
| STOP: | Leave patient bed/chair OR return to preparation area. |
|  | |
| START: | Go to preparation area. |
| Remove second pre-medication from packaging, go to patient bed/chair, remove empty bag (from previous pre-medication), connect next medication to patient IV line. | |
| STOP: | Leave patient bed/chair OR return to preparation area.  [Task repeated for third/fourth, etc., IV pre-medication] |
|  | |
| START: | [When machine beeps] Go to preparation area. |
| Remove empty bag from last pre-medication. | |
| STOP: | Leave patient bed/chair OR return to preparation area. |
| **Prepare for Rituximab Infusion and Infusion Initiation** | |
| START: | Go to preparation area. |
| Open bag and take out rituximab, verify if medication prescription corresponds with information on bag. | |
| If needed, have a second nurse check prescription. | |
| STOP: | Put bag in cupboard space reserved for patient. |
|  | |
| START: | Take bag and hang next to patient bed/chair. |
| Connect bag with infusion line and program infusion pump for initial infusion rate and initiate infusion. | |
| STOP: | Leave patient bed/chair. |
| **Dose Escalation/Patient Monitoring During Infusion** | |
| Monitoring # (this process is repeated for each monitoring event required) | |
| START: | [When machine beeps] Go to patient bed/chair. |
| Check patient. | |
| Adjust the infusion rate. | |
| STOP: | Leave patient bed/chair. |
| **Disconnect Infusion/Flush Infusion Line/Dispose of Materials** | |
| Flush via infusion | |
| START: | [When infusion completed] Collect NaCl solution and consumables. Go to patient bed/chair. |
| Disconnect rituximab bag from IV line, connect NaCl solution for flushing IV line via infusion. | |
| Discard used rituximab bag in special bin. | |
| STOP: | Leave patient bed/chair. |
|  | |
| START: | Go to patient bed/chair. |
| Close infusion pump, disconnect from patient, remove NaCl bag and giving set/infusion line and discard. | |
| *[if permanent line]* | Add heparin in the portacath/permanent reservoir. |
| *[if peripheral access]* | Remove peripheral catheter, discard catheter OR add heparin (if other infusions are expected on the same day or next day). |
| STOP: | Leave patient bed/chair. |
| Flush via direct injection/bolus | |
| START: | [When infusion completed] Collect NaCl solution and consumables. Go to patient bed/chair. |
| Disconnect rituximab bag from IV line, administer NaCl solution for flushing IV line via direct injection/bolus. | |
| Discard used rituximab bag in special bin. | |
| Close infusion pump, disconnect from patient, remove NaCl solution and giving set/infusion line and discard. | |
| *[if permanent line]* | Add heparin in the portacath/permanent reservoir. |
| *[if peripheral access]* | Remove peripheral catheter, discard catheter OR add heparin (if other infusions are expected on the same day or next day). |
| STOP: | Leave patient bed/chair. |
| **Patient Monitoring Post-infusion** | |
| Monitoring # (this process is repeated for each monitoring event required) | |
| START: | Go to patient bed/chair. |
| Check patient complaints and vital signs (blood pressure and pulse rate). | |
| STOP: | Leave patient bed/chair. |
