# Supplementary material for: Time Savings with Rituximab Subcutaneous Injection versus Rituximab Intravenous Infusion: A Time and Motion Study in Eight Countries
Source: PLoS One. 2016 Jun 30;11(6):e0157957. doi: 10.1371/journal.pone.0157957 (PMC4928781; doi:10.1371/journal.pone.0157957)
Supplement: S2 Table — IV, intravenous. (DOCX) [file pone.0157957.s002.docx]

**S2 Table. Pharmacy Generic Observation Form.**

| **IV Preparation in Pharmacy** | |
| --- | --- |
| START: | Take prescription and treatment order. |
| *[if required]* | Generate worksheet and prepare vials. |
| Go to the fridge. Gather all consumables and rituximab vial(s) to prepare the infusion; check expiry date and vial condition. | |
| STOP: | Bring consumables and rituximab vial(s) to aseptic preparation area. |
|  | |
| START: | Pick up rituximab vial(s) and consumables in aseptic preparation area. |
| Prepare a syringe to withdraw the appropriate amount of solution from the vial(s) and dilute the solution with NaCl solution. | |
| STOP: | Leave prepared rituximab bag ready for labelling/sign-off. |
|  | |
| START: | Collect prepared rituximab bag from aseptic preparation area. |
| Label drugs and receive final sign off and release of infusion. | |
| *[if required]* | Complete any forms/documents. |
| STOP: | Rituximab bag is left ready for collection. |
| **SC Preparation in Pharmacy** | |
| START: | Take prescription and treatment order. |
| *[if required]* | Generate worksheet and prepare vials. |
| Go to fridge. Gather all consumables and rituximab vial(s) to prepare the syringe; check expiry date and vial condition. | |
| STOP: | Bring consumables and rituximab vial(s) into aseptic preparation area. |
|  | |
| START: | Pick up rituximab vial(s) and consumables in aseptic preparation area. |
| Prepare a syringe to withdraw the appropriate amount of solution from the vial(s) and draw it into the syringe. | |
| STOP: | Leave prepared rituximab syringe ready for labelling/sign-off. |
|  | |
| START: | Collect prepared rituximab syringe from aseptic preparation area. |
| Double check prescription (batch number) and visually inspect the syringe. Provide final sign-off. Rituximab syringe is boxed up and sealed. | |
| STOP: | Rituximab syringe is left ready for collection. |
