# Supplementary material for: Time Savings with Rituximab Subcutaneous Injection versus Rituximab Intravenous Infusion: A Time and Motion Study in Eight Countries
Source: PLoS One. 2016 Jun 30;11(6):e0157957. doi: 10.1371/journal.pone.0157957 (PMC4928781; doi:10.1371/journal.pone.0157957)
Supplement: S3 Table — IM, intramuscular; IV, intravenous; SC, subcutaneous. (DOCX) [file pone.0157957.s003.docx]

**S3 Table. Rituximab SC Generic Observation Form.**

| **Pre-medication Administration** | |
| --- | --- |
| If pre-medications are conducted as a single activity. | |
| START: | Go to preparation area and collect all required pre-medications. Go to patient bed/chair. |
| *[oral drug]* | Medication and something to drink given to patient and uptake is controlled. |
| *[IV drug]* | Remove pre-medication from packaging; administer the medication via bolus injection. |
| *[IM or SC drug]* | Remove pre-medication from packaging; administer the medication via direct injection. |
| *[if required]* | Administer further pre-medications. |
| *[after final pre-medication administration]* | Flush line with NaCl solution via bolus injection. |
| STOP: | Leave patient bed/chair OR return to preparation area. |
| If pre-medication administration is conducted as a sequence of separate activities. | |
| START: | Go to preparation area. |
| Remove first pre-medication from packaging, go to patient bed/chair and connect medication to patient IV line. | |
| STOP: | Leave patient bed/chair OR return to preparation area |
|  | |
| START: | Go to preparation area. |
| Remove second pre-medication from packaging, go to patient bed/chair, remove empty bag (from previous pre-medication), connect next medication to patient IV line. | |
| STOP: | Leave patient bed/chair OR return to preparation area.  [Task repeated for third/fourth etc., IV pre-medication] |
|  | |
| START: | [When machine beeps] Go to preparation area. |
| Remove empty bag from last pre-medication. | |
| STOP: | Leave patient bed/chair OR return to preparation area. |
| **Bringing Rituximab to Patient Bed/Chair and Injection Administration** | |
| START: | Go to preparation area. |
| Open bag and take out rituximab syringe, verify if medication prescription corresponds with information on medication, prepare syringe. | |
| STOP: | Put syringe in cupboard space reserved for patient. |
|  | |
| START: | Take injection from cupboard space. |
| Go to patient bed/chair, initiate injection (insert needle using sterile technique into subcutaneous tissue of the abdomen). | |
| Monitor for any immediate signs of adverse reaction. | |
| STOP: | Discard empty syringe. |
| **Patient Monitoring Post-injection** | |
| Monitoring # (this process is repeated for each monitoring event required) | |
| START: | Go to patient bed/chair. |
| Monitor for any immediate signs of adverse reaction. | |
| STOP: | Leave the patient bed/chair |
