# Supplementary material for: Time Savings with Rituximab Subcutaneous Injection versus Rituximab Intravenous Infusion: A Time and Motion Study in Eight Countries
Source: PLoS One. 2016 Jun 30;11(6):e0157957. doi: 10.1371/journal.pone.0157957 (PMC4928781; doi:10.1371/journal.pone.0157957)
Supplement: S4 Table — DPA: drug preparation area; HCP: healthcare professional; IV: intravenous; SC: subcutaneous. *Indicates results that were derived from the random intercept model; all other results were derived from the standard regression analysis (gamma was the best fitting distribution in almost all cases). (DOCX) [file pone.0157957.s004.docx]

**S4 Table. Active HCP Time in Treatment Room and DPA, by Task and by Country (per session).**

| **Active HCP Time in Treatment Room and DPA, by Task, per Session (minutes)** | **Austria** | | **Brazil** | | **France** | | **Italy** | | **Russia** | | **Slovenia** | | **Spain** | | **UK** | | |
| --- | --- | --- | --- | --- | --- | --- | --- | --- | --- | --- | --- | --- | --- | --- | --- | --- | --- |
|  | **IV** | **SC** | **IV** | **SC** | **IV** | **SC** | **IV** | **SC** | **IV** | **SC** | **IV** | **SC** | **IV** | **SC** | **IV** | **SC** | |
| **Install Venous Catheter/Line Flushing** | 4.7 | - | 6.7 | - | 11.7* | - | 3.8 | - | 3.8* | - | 9.1 | - | 2.7 | - | 10.5 | - | |
| **Pre-medication Administration** | 3.4 | 2.1 | 2.4 | 2.0 | 5.2* | 4.9* | 4.5 | 3.3 | 2.5* | 1.6 | 1.2 | 1.1 | 3.0 | 2.9 | 8.3 | 2.1 | |
| **Bringing Rituximab Bag to Patient Bed/Chair** | - | - | - | - | 1.1* | - | 2.9 | 2.2 | - | - | - | - | - | - | - | - | |
| **Infusion Initiation/ Injection Administration** | 1.1 | 6.3 | 3.3 | 6.0 | 4.1* | 8.9* | 2.5 | 7.2 | 3.3* | 7.2 | 1.1 | 6.7 | 1.8 | 8.0 | 12.8 | | 12.9 |
| **Patient Monitoring During Infusion/ Injection** | 0.9 | - | 1.8 | - | 2.3* | - | 8.3 | - | 8.4* | - | 0.3 | - | 3.5 | - | 3.7 | - | |
| **Disconnect Infusion/Flush Line/Dispose of Materials** | 2.0 | - | 2.1 | - | 5.5* | - | 6.5 | - | 2.5* | - | 3.3 | - | 1.8 | - | 5.3 | - | |
| **Patient Monitoring Post-Infusion/Injection** | 0.1 | 0.3 | 0.1 | 1.2 | 0.2* | 0.3 | 5.7 | 7.2 | 4.5* | 2.1 | 0.1 | - | 0.8 | 2.4 | - | 0.1 | |
| **DPA Tasks (composite)** | 10.9 | 7.4 | 9.3 | 4.8 | 11.9* | 9.7* | 6.8 | 4.5 | 5.5* | 1.8 | 3.7 | 1.6 | 12.7 | 5.9 | 38.9 | 33.3 | |
